# Susceptibility of Faba Bean (*Vicia faba* L.) to Heat Stress During Floral Development and Anthesis

**DOI:** 10.1111/jac.12172

**Published:** 2016-03-21

**Authors:** J. Bishop, S. G. Potts, H. E. Jones

**Affiliations:** ^1^ School of Agriculture Policy & Development University of Reading Reading Berkshire UK

**Keywords:** abiotic stress, climate change, faba bean, heat stress, *Vicia faba*, yield

## Abstract

Experiments were conducted over 2 years to quantify the response of faba bean (*Vicia faba* L.) to heat stress. Potted winter faba bean plants (cv. Wizard) were exposed to temperature treatments (18/10; 22/14; 26/18; 30/22; 34/26 °C day/night) for 5 days during floral development and anthesis. Developmental stages of all flowers were scored prior to stress, plants were grown in exclusion from insect pollinators to prevent pollen movement between flowers, and yield was harvested at an individual pod scale, enabling effects of heat stress to be investigated at a high resolution. Susceptibility to stress differed between floral stages; flowers were most affected during initial green‐bud stages. Yield and pollen germination of flowers present before stress showed threshold relationships to stress, with lethal temperatures (*t*_50_) *˜*28 °C and ~32 °C, while whole plant yield showed a linear negative relationship to stress with high plasticity in yield allocation, such that yield lost at lower nodes was partially compensated at higher nodal positions. Faba bean has many beneficial attributes for sustainable modern cropping systems but these results suggest that yield will be limited by projected climate change, necessitating the development of heat tolerant cultivars, or improved resilience by other mechanisms such as earlier flowering times.

## Introduction

Future global crop production faces interacting challenges; with an increasing global population, more food is required, but with climate change and associated extreme weather conditions (Hansen et al. 2012), it is becoming more difficult to produce food (Porter et al. 2014). Crop yield is particularly susceptible to adverse weather conditions during floral development and anthesis (Luo 2011), a relatively short stage crucial to yield production that is highly susceptible to short periods of high temperature and/or drought. To allow improvement of crop–climate models, planning of adaptation measures (such as agronomic changes), and breeding of new genotypes capable of tolerating or avoiding projected stresses, it is important to thoroughly quantify the response of crops to heat stress (Siebert and Ewert 2014). Through controlled environment studies, the impact of heat stress during floral development and anthesis on crop yield has now been quantified for many species (*e.g*. Hedhly 2011, Luo 2011), allowing extreme weather events to be incorporated into crop‐climate models (*e.g*. Deryng et al. 2014). However, the response of faba bean (*Vicia faba* L.) to heat stress during floral development and anthesis has not been previously investigated. Faba bean may be particularly valuable in the drive towards increased food production and sustainable intensification (Pretty and Bharucha 2014). It is capable of yield production independent of external mineral nitrogen inputs and has multiple benefits to modern cropping systems including being a useful break crop (Köpke and Nemecek 2010), while providing a valuable source of protein (Crépon et al. 2010).

More is known about the responses of other grain legumes to heat stress. Common bean (*Phaseolous vulgaris* L.) genotypes showed reduced yield mass, seed set and pod set when exposed to day temperatures of 32 °C during floral development (Gross and Kigel 1994, Porch and Jahn 2001, Suzuki et al. 2001). Similar responses to heat stress have been measured in pea (*Pisum sativum* L.) at 30 °C (McDonald and Paulsen 1997) and cowpea (*Vigna unguiculata* L.) at 33 °C (Ahmed et al. 1992). Yield reductions with heat stress are often caused by a combination of reduced pod and seed set, highlighting issues with fertilization following the stress event; with the exception of McDonald and Paulsen (1997), the above studies also recorded corresponding reductions in anther dehiscence and pollen viability. Above optimal temperatures can also accelerate floral development, and there is evidence from research in wheat (*Triticum aestivum* L.) that this may negatively impact on yield, due to an increased rate at which resources are required by developing seeds, to a point where resource‐deficit occurs (González et al. 2011).

In experiments conducted over 2 years, plants were subjected to a range of temperatures to quantify the response of faba bean to heat stress during floral development and anthesis. This study has focused on whole plant responses to promote wider applicability of its findings, measuring whole plant yield mass, seed set, pod set, yield distributions and corresponding changes in pollen germination. Given the lack of information regarding faba bean responses to heat stress, the aim of this research was to investigate several key questions: (i) Is faba bean susceptible to heat stress within the temperature range known to damage other crops? (ii) What are the consequences of heat stress for faba bean yield? (iii) What are the mechanisms of yield reductions, and can yield reductions be linked to gametophyte damage as in other species?

## Methods

### Growing conditions and experimental design

Pot experiments were conducted in 2013 and 2014 at the Plant Environment Laboratory (now succeeded by the Crop and Environment Laboratory), University of Reading, UK (51 27′N latitude, 00 56′W longitude) to investigate the response of winter faba bean (*Vicia faba* L. cv. Wizard) to heat stress during floral development and anthesis. The synthetic cultivar Wizard (Wherry & Sons Ltd.) has wide current usage in the UK and is suitable to cultivation in a variety of environments (Flores et al. 2012).

All experiments used plastic pots (180 mm diameter; 4 l volume) containing 2 : 1 : 2 : 0.5 of vermiculite: sand: gravel: compost mixed with Osmocote slow‐release granules (2 kg m^−3^) containing a ratio of 15 : 11 : 13 : 2 of N : P_2_O_5_ : K_2_O : MgO. Three seeds were sown per pot, which allowed thinning to one plant per pot when three leaf pairs were unfolded (BBCH 13, uniform decimal code for plant growth; Lancashire et al. 1991). Pots were maintained in a polytunnel until four leaf pairs were unfolded on the majority of main stems (BBCH 14), at which point they were moved outside and randomly distributed in experimental cages. The experiments were conducted as part of a larger study investigating interactions between heat stress and insect pollination. Cages were used to prevent floral visitation and pollen movement between flowers by insect pollinators. Cages were constructed from 1.33 mm^2^ nylon mesh (WM16, Wondermesh Ltd.), suspended by shock cord from a metal frame and sealed between wooden boards at their base. In 2013, a single cage was used with measurements 12.5 × 2.5 × 2.5 m, and it was ventilated by a 30‐cm fan with air distributed along the length of the cage via polythene tubing pierced at regular intervals. In 2014, five separate unventilated cages were used to increase replication for pollination treatments; each measured 2.5 × 2.5 × 2.5 m. In both years, plants were watered daily by drip‐irrigation to maintain field capacity throughout the experiment.

Heat stress treatments (18, 22, 26, 30, 34 °C day temperature) were chosen to measure responses over a range of temperatures. They comprised transferring plants to 1.37 × 1.47 m^2^ Saxcil growth cabinets for a duration of 5 days (summary of developmental stages prior to stress provided in Fig. 3 and Table S1, supplementary information). The photoperiod lasted 16 h, night temperatures were 8 °C below day temperatures, and the transition between night and day temperatures took approximately 15 min. Light levels were maintained at 650 μmol photon m^−2^ s^−1^; relative humidity at 80 ± 20 % in 2013 and 85 ± 15 % in 2014; CO_2_ at 385 mg l^−1^. Irrigation to field capacity was continued during heat stress. Temperature was measured by a thermocouple at 32 cm from the base of each cabinet.

In 2013, three consecutive replicate experiments were conducted over a period of 18 days. Each experiment used 95 plants, from a group of 285 plants that were sown on 11 January and randomly pre‐assigned to temperature treatments. To improve comparability between replicate experiments, plants were manually assigned to replicates according to their developmental stage, with the most advanced plants assigned to the first replicate experiment (Fig. 3; Table S1, supplementary information). For each replicate experiment, 19 plants were transferred from the pollination cage to each growth cabinet at midday, left for 5 days, then removed and returned back to the cage. Growth cabinet temperatures were randomly assigned between experiments, and conditions were monitored throughout the treatment duration. Plants were removed from cabinets at midday, allowing 24 h to re‐assign cabinet temperature treatments before the next replicate set of plants was added on the following day. In 2014, 100 plants were sown on 13 January and were used in a single additional replicate experiment using 20 plants per cabinet, with one cabinet used for each of the five temperatures.

In the morning prior to heat stress treatment, all plants in a replicate experiment were scored for floral development. On all plants, individual flowers at the first, second, third and last raceme positions at each visible floral node were non‐destructively scored, and assigned to a developmental scale (Table 1). In 2013, flowers on 15 plants per experiment were also scored following stress (n =* *3265). The floral development scale was based on relative sizes of the calyx and corolla and built upon an existing scale for faba bean (Osborne et al. 1997, Kambal 1969). Ad hoc dissections performed on spare plants confirmed that attributes of pollen development were shared with equivalent stages assigned by Osborne et al. (1997) which were based upon a different cultivar.

**Table 1:** Floral development stages for faba bean, adapted from Osborne et al. (1997)

Stage	Description
0	Flower not present (or inaccessible to score non‐destructively)
1	Calyx < 5 mm long
2	Calyx < 10 mm long
3	Corolla first visible
4	Corolla ≤ ¼ total flower length
5	Corolla ≤ ½ total flower length
6	Corolla > length of calyx and still furled
7	Corolla unfurling along bottom edge (standard petal unfurling)
8	Standard petal becoming erect (<45° angle away from wing petals)
9	Standard petal erect (≥45° angle away from wing petals)
10	Standard petal wilted/flower wilted

Yield parameters were assessed when plants had reached senescence (stems and pods uniformly black). All pods on experimental plants were individually harvested with their nodal‐ and racemal‐position recorded, enabling harvest records to be matched with pre‐stress floral development stages, such that interactions of pre‐stress stages and heat stress on yield parameters could be investigated. Pods were oven‐dried at 80 °C until dry mass was constant before recording total pod mass, bean mass and bean number per pod. There were slight sampling differences between experimental years; fewer small beans were included in counts for 2014. Bean size records measured using WINDIAS image analysis software were used to derive a conservative bean number parameter with small beans (area <50 mm^2^) removed from counts in both years.

### Pollen germination testing

In 2013, an additional group of 45 plants (15 per experiment) was used to assess pollen damage after exposure to heat stress treatments (Table S2, supplementary information). Flowers scored at development stages 1 to 6 (Table 1) before stress were destructively sampled after reaching stage 7 (which corresponded with anther dehiscence; Osborne et al. 1997), and before reaching stage 10 (senescence). Preference was given to flowers at lower nodal and racemal positions at stages 7 and 8. Pollen *in vitro* germinability was assessed using a methodology adapted from Dafni et al. (2005); pollen of individual flowers was added to 10 *μ*l droplets of nutrient medium in modified petri dishes, allowed to germinate for 24 h at room temperature and proportion germination counted by light microscopy. The nutrient medium was optimised to faba bean and prepared by adding sucrose (27.5 g sucrose per 100 g final solution) to a 1 : 1 mixture by volume of 2 × 10^−3^ m H_3_BO_3_ and 6 × 10^−3^ m Ca(NO_3_)_2_. Samples were refrigerated at 0–5 °C until examination. Droplets were stained with 5 *μ*l methylene blue (1 % in water), and pollen grains counted as germinated if the pollen tube was greater than or equal to the diameter of the pollen grain. For each sample, a total of five counts at 80× magnification were made at different positions on the slide, and these individual counts were summed to generate totals for each sample.

### Statistical analysis

Statistical analysis was performed using the software R (version 3.1.2; R Core Team 2014). In all analyses, 5‐day cabinet ‘runs’ were treated as the experimental unit; for example, per‐plant yield parameters were calculated by summing individual pod weights for each plant, from which mean per‐plant yield was calculated at each heat stress temperature (5 temperature levels × 4 replicate experiments). Year and replicate experiment were tested as fixed effects in all analyses including in an interaction term with heat stress temperature, to control for temporal variability between replicate experiments or growing seasons. The five individual cages used in 2014 were treated as a single cage, as the main treatment applied was heat stress temperature. Relationships of parameters to heat stress temperature were modelled by linear and non‐linear regression. Quadratic terms were tested to assess curvature as an initial indicator of threshold relationships (*e.g*. Craufurd et al. 2013). When appropriate, a 3‐parameter logistic model (*y* = *a*/(1 + *b*e^−*c*x^), where *a* is the asymptotic value, *b* is the temperature where the dependent parameter is *a*/2, and *c* is the scale, was fitted and favoured over the linear model with quadratic term if model explanatory power was significantly improved (measured by Akaike's Information Criterion corrected for small sample sizes, AICc).

To assess interactions between heat stress temperature and pre‐stress floral developmental stage on yield, the total yield mass produced by pods from flowers of each stage (1 to 6) was calculated for each plant, to produce a mean per‐plant yield contribution from each floral development stage (6 stages × 5 temperature levels × 4 replicate experiments). Yield allocation per node per plant was modelled by generalised additive models which fitted a separate smoothed line for each temperature treatment, of each replicate experiment. An arbitrary limit of 5 basis dimensions was set, to force simple model fits that were readily comparable, and the node at which the highest point of each fitted line occurred was then regressed against temperature.

To establish the effect of treatments on each yield parameter, maximal models (containing temperature, a quadratic term for temperature, year or replicate experiment in an interaction with temperature, and where appropriate, floral developmental stage) were simplified using manual stepwise backwards selection, and models were further simplified by grouping categorical factor levels with similar model predicted estimates (Crawley 2013). Parameters were dropped if P* *>* *0.05, provided that model fit (adjusted r^2^) or residual performance were not compromised. Model residuals were checked for normality and heteroscedasticity; subset yield and pollen germination were log‐transformed, and floral‐scale yield was square‐root transformed to improve model fit.

## Results

### Whole plant yield

**Figure 1:**
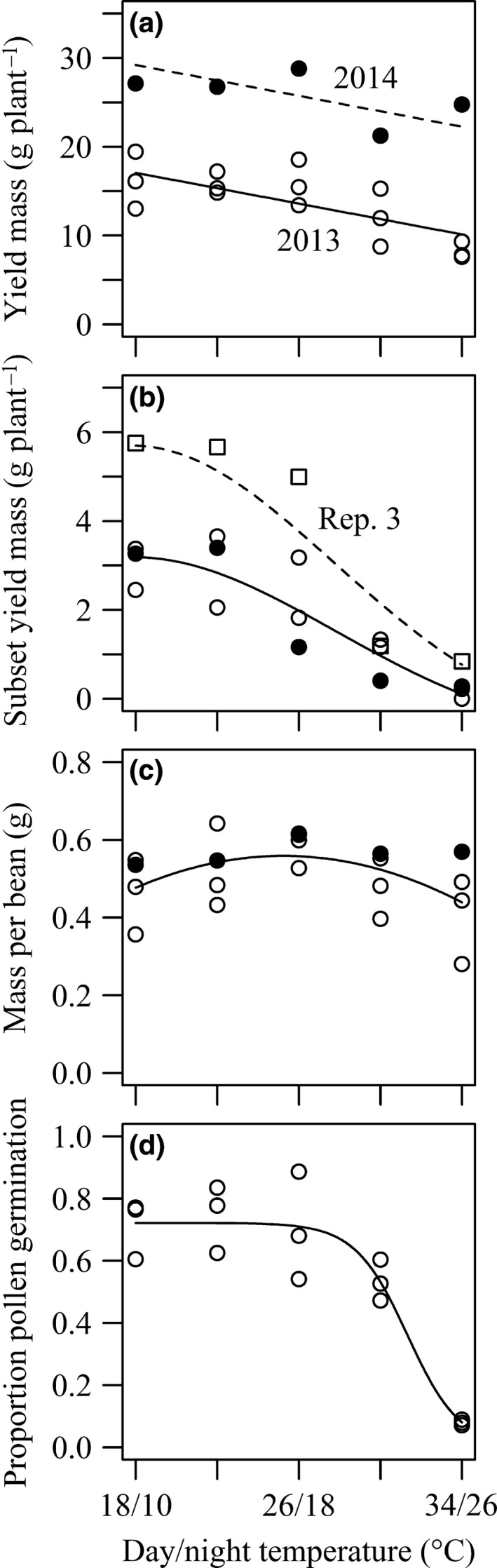
Responses of faba bean plants exposed to heat stress temperature treatments for 5 days during floral development and anthesis. Presented points are means of each parameter at each heat stress temperature treatment for up to four replicate experiments; open points indicate 2013 (3 replicate experiments); filled points indicate 2014 (1 additional experiment); and presented lines are best model fits from linear regressions. Figures represent parameters: (a) whole plant yield mass; (b) subset plant yield mass defined as mean per‐plant yield of pods from flowers that were present prior to stress, square points are replicate 3; (c) individual bean mass, the line represents both years; and (d) per‐plant proportion *in vitro* pollen germination.

The total yield mass produced by individual plants decreased from the lowest to the highest temperature treatment (Fig. 1A). This decrease was best modelled by a straight line with slope −0.434 (95%CI (95% Confidence Interval) −0.652 to −0.216), estimating an average reduction in per plant yield of 6.9 g (95%CI 10.4 g to 3.5 g) from the lowest to the highest heat stress treatment. This response of yield mass to temperature did not vary between years of experimentation, an interaction term between heat and year did not improve model fit (P = 0.308); although absolute plant yield mass was on average 12.1 g (95%CI 9.3 g to 15.0 g) higher in 2014. While data presented in Fig. 1a may suggest a possible threshold relationship of per‐plant yield mass to temperature, a quadratic term tested in the model did not improve model fit (P = 0.088). The number of beans produced by individual plants was analysed to assess how self‐fertilization was modified by stress (Table 2). Bean number per plant decreased by an average of one bean for every degree increase in temperature (95%CI −1.561 to −0.533) in both years, and plants produced on average 21.5 (95%CI 15.102–27.924) more beans in 2014. Pod number per plant (Table 2) had a different response to the increase in temperature depending on the year (interaction term; P = 0.028); on average, one pod per plant was lost for a 3.8 °C temperature increase in 2013 (95%CI 2.3–10.8), and gained for an 8 °C increase in 2014 (95%CI difference between year slopes 0.048–0.728). Individual plants varied dramatically in their yield performance, and this variability (coefficient of variation; mean/standard deviation) did not significantly change between heat stress temperature treatments, years or replicate experiments.

**Table 2:** Parameter estimates of the minimal‐adequate model for each yield parameter. Base levels are indicated where applicable, estimates provided for other treatment levels are of the difference from base estimates. Parameter estimates remain transformed where indicated

Parameter	Estimate	Standard error	*t*‐value	P‐value
Model: yield mass per plant ~ heat + year	Adjusted r^2^: 0.835			n* *=* *20
Intercept (base; year 1)	24.885	2.771	8.980	**<0.001**
Intercept (year 2)	12.135	1.350	8.986	**<0.001**
Slope	−0.434	0.103	−4.198	**<0.001**
Model: bean number per plant ~ heat + year	Adjusted r^2^: 0.796			n = 19
Intercept (base; year 1)	51.246	6.629	7.731	**<0.001**
Intercept (year 2)	21.513	3.024	7.114	**<0.001**
Slope	−1.047	3.024	−4.316	**<0.001**
Model: pod number per plant ~ heat * year	Adjusted r^2^: 0.795			n = 20
Intercept (base; year 1)	18.996	2.135	8.900	**<0.001**
Intercept (year 2)	−2.777	4.269	−0.650	<0.525
Slope (base; year 1)	−0.263	0.080	−3.278	**<0.005**
Slope (year 2)	0.388	0.160	2.418	**<0.028**
Model: log(subset yield mass+1) ~ heat + heat^2^ + replicate	Adjusted r^2^: 0.860			n = 20
Intercept (base; rep. 1,2 and 4)	−0.188	1.190	−0.158	<0.877
Intercept (rep. 3)	0.469	0.112	4.186	**<0.001**
Slope	0.182	0.947	1.921	<0.073
Quadratic	−0.005	0.002	−2.813	**<0.013**
Model: mass per bean ~ heat + heat^2^	Adjusted r^2^: 0.149			n = 20
Intercept	−0.437	0.465	−0.938	<0.362
Slope	0.079	0.037	2.128	**<0.048**
Quadratic	−0.002	0.001	−2.200	**<0.042**
Model: log(pollen germination+1) ~ *a*/(1 + *b*e^−*c*heat^)	Adjusted r^2^: N/A			n = 15
*a*	0.543	0.020	27.196	**<0.001**
*b*	31.671	0.414	76.423	**<0.001**
*c*	−1.294	0.266	−4.858	**<0.001**
Model: √floral stage yield ~ heat * stage + heat^2^ + replicate	Adjusted r^2^: 0.692			n = 120
Intercept (base; stage 1)	1.435	0.546	2.630	**0.010**
Intercept (stage 2)	−0.721	0.333	−2.168	**0.034**
Intercept (stages 3–6)	−2.037	0.263	−7.745	**<0.001**
Slope (base; stage 1)	0.023	0.041	0.574	0.567
Slope (stage 2)	0.020	0.013	1.608	0.111
Slope (stages 3–6)	0.056	0.010	5.689	**<0.001**
Quadratic	−0.002	0.001	−2.477	**0.015**
Intercept (reps. 2 and 3)	0.153	0.050	3.068	**0.003**
Intercept (rep. 4)	0.025	0.058	0.427	0.670
Model: node with yield maxima ~ heat + year	Adjusted r^2^: 0.934			n ***= ***19
Intercept (year 1)	−1.205	0.785	−1.536	0.144
Intercept (year 2)	−1.317	0.385	−3.420	**<0.004**
Slope	0.456	0.029	15.616	**<0.001**

Bold values are significant to P* *<* *0.05

### Subset plant yield

In addition to whole plant scale yield parameters, it was possible to investigate responses of flowers most likely to be directly affected by heat stress treatments. Floral developmental stages recorded immediately prior to heat stress allowed a separate parameter to be calculated that included the yield of pods set from flowers that were scored as present (floral developmental stage ≥ 1) prior to heat stress. Subset yield mass was found to respond differently to temperature from the lowest to the highest heat stress temperature treatment; declines in yield were steeper between the higher temperature treatments (Fig. 1b). This curvature was quantified by incorporating a quadratic term for heat in the regression model, which improved model fit (P =* *0.013) and *t*_50_ was established as *˜*28 °C. The main variability in subset yield was found to occur between replicate experiments in 2013, rather than between years. Yield was higher in replicate 3; all other replicate experiments had similar yields and were treated as a single factor level, improving model fit (P =* *0.329).

### Individual bean mass

To investigate whether reductions in whole plant yield mass were more likely due to either reductions in the absolute number of beans (indicating either reduced fertilization or increased abortion), or to changes in bean size, the parameter of mass per bean was assessed (Fig. 1c). The mass of individual beans did not show a negative relationship to temperature and remained within the range of 0.44 g to 0.56 g (95%CI min = 0.35 g; max = 0.62 g) in the tested temperature range. The best model included a humped response to temperature (quadratic term; P = 0.042) where mass per bean was highest at *˜*25 °C, variation between years was not significant (P = 0.078). The best model did not provide a good fit to the data, with an r^2^ value of 0.149.

### Pollen germination

**Figure 2:**
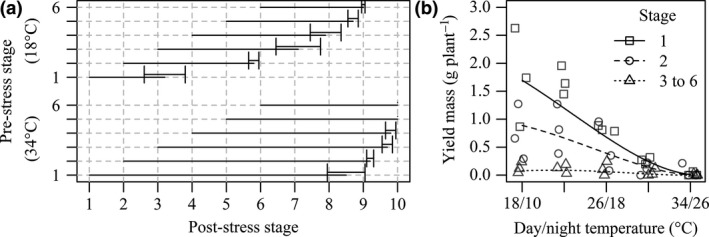
(a) Floral development stage scores before and after heat stress treatment temperatures 18 °C and 34 °C. Presented values are mean post‐stress stage of flowers scored at each pre‐stress stage from 3 replicate experiments in 2013. Error bars are one standard deviation of the mean. (b) Interactions of pre‐stress floral development stage and heat stress for 5 days during floral development and anthesis on yield of faba bean plants. Presented data are mean mass produced by pods from flowers of different pre‐stress floral development stages, at each heat stress treatment across four replicate experiments. Presented lines represent separate development stage category predictions averaged across replicate experiments.

Direct damage to those flowers present prior to heat stress treatment was assessed by measuring *in vitro* pollen germination on a per‐plant scale, from a separate subset of 45 plants in 2013. Pollen germinability showed a threshold effect within the temperature range tested (Fig. 1d), being best modelled by a non‐linear 3‐parameter logistic regression (*y* = *a*/(1 + *b*e^−*c*x^)) (compared to linear model with quadratic term ΔAICc = 4.148). This fitted a flat response until a lethal temperature, after which there was a decline with slope factor (*c*) of −0.710 (95%CI −0.847 to −0.510), with 50 % pollen germination estimated to be lost at approximately 32 °C (*b = *31.671 °C; 95%CI 30.768 °C to 32.574 °C). Pollen germination was not complete at control temperatures (antilogged model‐estimated asymptote *a *=* *0.721; 95%CI 0.649–0.799), suggesting either an inadequate germination test or incomplete germination. The number of flowers at a developmental stage suitable for testing (Table S2, supplementary information) decreased from the lowest to the highest temperature treatment, due to increased floral development rate (Fig. 2a). To compensate for this, only the pollen germinability of flowers with pre‐stress developmental stages between 1 and 3 (Table 1) was analysed.

### Damage on a floral scale

Developmental stage records of individual flowers (Table 1) allowed investigation of how the response of yield to heat stress may vary at the scale of individual flowers, according to their developmental stage prior to stress. Flowers continued to develop during the 5‐day heat stress treatment, such that stress exposure occurred at later developmental stages in addition to the recorded ‘pre‐stress’ stage (Fig. 2a). However, these stages represent distinct categories; for example, only flowers scored at stage 1 prior to stress were exposed to stress at stage 1, and stage 3 flowers did not receive stress at stages 1 or 2. Yield of flowers scored at different pre‐stress floral developmental stages showed contrasting responses to increasing heat stress temperature, with a significant interaction between developmental stage and heat (P* *<* *0.001). Floral developmental stages were best modelled using three categories with similar responses to temperature (compared to model including 6 separate stages; P =* *0.499); these were stage 1, stage 2 and stages 3 to 6 combined in a single category. Stages 1 and 2 showed greater susceptibility to heat stress than other stages, with significantly steeper declines in yield from the lowest to the highest heat stress treatment (Fig. 2b). The main variability in yield was found to occur between replicate experiments, with higher yields in replicates two and three of 2013, grouping these replicate experiments did not impair model fit (P = 0.219). It was not possible to use the measurements of pollen germination at the scale of individual floral stages due to the accelerating effect of higher temperatures on floral development (Fig. 2a). There were insufficient numbers of suitable flowers for pollen germination testing (post‐stress stage below 10) from higher temperature treatments, or more advanced pre‐stress stages. An analysis using only data from flowers of pre‐stress stages 1 to 3 found no significant interaction between stage and heat stress treatment on proportion pollen germination.

### Yield distributions

**Figure 3:**
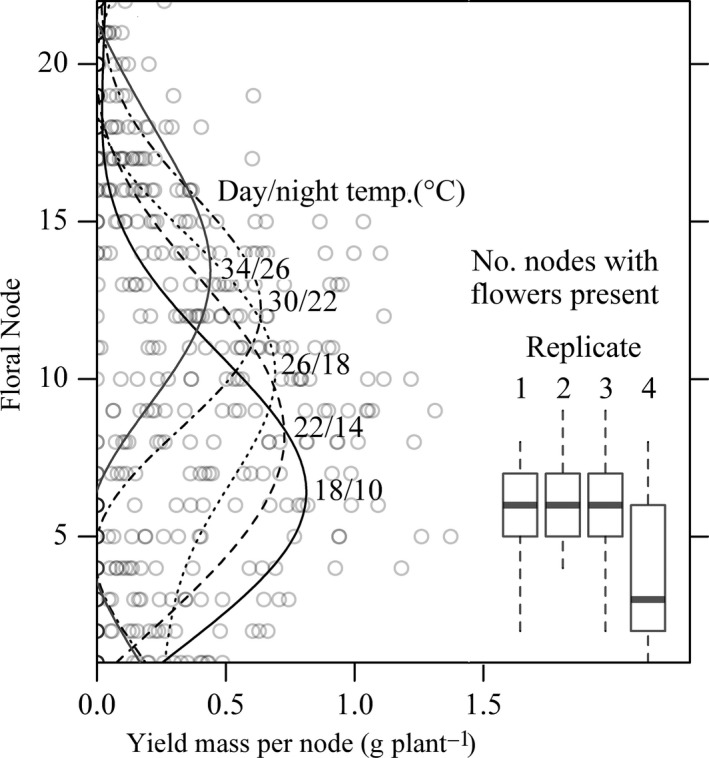
Response of primary stem yield allocation in faba bean to heat stress temperature, in relation to the location of flowers prior to treatments. Presented data points are average yield per node per plant for each heat stress temperature of four replicate experiments. Boxplots show the number of floral nodes with flowers present (counts include nodes with un‐opened flowers, *e.g*. stage ≥ 1) on primary stems prior to temperature treatments in each replicate experiment. Presented lines are fits of generalised additive models at each temperature (averaged across 4 replicate experiments).

Using individual records of pods harvested from main stems, it was possible to assess how heat stress treatments changed the distribution of yield within plants (Fig. 3). The floral node position with the highest model‐estimated yield increased with temperature (away from those flowers present prior to stress; Fig. 3) at a slope of 0.456 (95%CI 0.394–0.518), equating to an upwards movement in yield allocation of 7.3 nodes (95%CI 6.3–8.3) from the lowest to the highest heat stress temperature (Table 2). Pod set was on average 1.3 nodes lower (95%CI −2.1 to −0.5) in 2014 (P = 0.015). The floral node position of one replicate (26 °C, replicate 3) was not included in the regression analysis as it was not possible to fit a comparable smoothed line.

## Discussion

Heat stress during floral development causes reductions in key yield parameters of faba bean. While there has been extensive work demonstrating the negative effects of drought stress in faba bean (Khan et al. 2010), the only previous work relating to heat stress has focused at initial vegetative growth stages (Hamada 2001, Oney and Tabur 2013). Heat stress impacts during reproductive development are often under‐researched relative to vegetative growth due to several difficulties in this area of study (Zinn et al. 2010, Hedhly 2011). It can be difficult to reliably identify key stages of reproductive development (Barber et al. 2015), reproductive development occurs over a short space of time, and to quantify yield effects requires long term experiments (Zinn et al. 2010). While field experiments may provide more realistic representations of the processes that occur in agricultural systems, they provide further challenges in their implementation; it is extremely difficult to control the magnitude of stress applied and to provide an adequate experimental control, and field conditions can increase random variation requiring larger, more expensive experiments.

The yield reductions of at least 24 %, measured between the lowest and highest temperature treatments, occurred within the temperature range known to damage other legume crops. Reductions in seed set, pod set and yield mass have been documented in common bean (*P. vulgaris*) following exposure to 32 °C day temperatures for 24 h (Suzuki et al. 2001), 5 days (Gross and Kigel 1994) and until maturity (Porch and Jahn 2001), and in pea (*P. sativum*) following exposure to 30 °C for 7 days (McDonald and Paulsen 1997). In this study, pod set was found to be a poor estimator for whole plant yield mass in faba bean, showing variability between years that did not correspond to changes in yield. The susceptibility of faba bean yield measured was also similar to other crops; temperatures of around 30 °C during floral development and anthesis elicit yield reductions in key cereal crops wheat, rice and maize (*e.g*. Hedhly 2011, Luo 2011). Although comparisons between crops are not straightforward, this similarity in response suggests that the modelling studies and yield projections for other crops could be reasonably translatable to faba bean, grown in equivalent conditions.

Mass per bean, which is interchangeable with thousand grain weight, an important quality parameter for growers, did not show a clear negative trend to increasing temperature. Mass per bean peaked at intermediate temperature treatments which were perhaps more conducive to faba bean growth; the optimal temperature for faba bean growth is around 22–23 °C (Patrick and Stoddard 2010). This absence of a negative relationship to temperature suggests that the recorded yield reductions were more likely due to issues with fertilization and reductions in bean number, than reductions in bean size. Many authors suggest that the development of gametophytes, and consequently fertilization, is an especially vulnerable stage within plant development (*e.g*. Craufurd et al. 2013); studies that have measured reductions in seed size have typically continued to expose plants to stress during grain filling (Prasad et al. 2003, Young et al. 2004).

The floral stages that were identified as most susceptible to heat stress are likely to coincide with gametophyte development in faba bean flowers. They occur well before stages that coincide with anther dehiscence (Kambal 1969, Osborne et al. 1997) and correspond to susceptible stages found in faba bean and other legume species. A study comparing floral stage susceptibility to hot winds in faba bean found that ‘pre‐corolla’ flowers were more susceptible to stress than older flowers (Bennell et al. 2007). In other legume species, authors have measured greater reductions in yield (and pollen viability) in flowers stressed earlier in their development (Monterroso and Wien 1990, Ahmed et al. 1992, Gross and Kigel 1994). It was not possible to attribute stress at different floral stages to changes in pollen viability; this would require a different study design with heat stress events of a shorter duration. However, as a simple assessment of sexual organ damage following heat stress, proportion *in vitro* pollen germination provided evidence of gametophyte damage on a plant scale and the derived lethal temperature (*t*_50_) of 32 °C enables comparison with other species. Previous work had suggested that faba bean may be more susceptible to (drought) stress compared to *P. sativum* (McDonald and Paulsen 1997). Similar reductions in pollen stainability have been measured at 32 °C in one *P. vulgaris* cultivar (Porch and Jahn 2001), and at average day temperatures of 27–28 °C in another (Suzuki et al. 2001). These studies highlight the variation that can occur between cultivars, experimental systems and floral development stages. In this experimental system, individual plant yields were very variable within treatments, as is to be expected in a synthetic cultivar due to high levels of heterozygosity (Stelling et al. 1994). This variability remained constant between temperature treatments and years and reduced the number of possible treatment combinations such as testing responses of different cultivars.

Pollen damage has been correlated to yield declines in many studies (*e.g*. Suzuki et al. 2001). In this study, large reductions in pollen germination were recorded, but at higher temperatures than those at which yield reductions occurred in similar flowers. This may have occurred because pollen germination was not a complete indicator of pollen viability; some damaged pollen may not have been released due to anther indehiscence, and pollen vegetative nuclei may have retained function while generative nuclei were damaged (Dafni et al. 2005). It could also be the case that female organs were damaged in this study. Female organs can be damaged to at least an equal extent by heat stress (Zinn et al. 2010), although male development has been shown to be more susceptible than female in some legumes (Monterroso and Wien 1990, Ahmed et al. 1992, Gross and Kigel 1994). Given the difference in response to pollen germination and yield to temperature treatments, proportion *in vitro* pollen germination is not a useful predictor of heat tolerance in faba bean.

There is a discrepancy between the straight line relationship to increasing heat stress temperature found for whole plant yield and the curved relationship found in yield from flowers that were present prior to heat stress. This could simply be due to insufficient statistical power to detect curvature, as a slight threshold effect is visible for whole plant yield. The discrepancy may also indicate that whole plant yields were buffered, through reallocation of resources to less damaged areas of the plants (*e.g*. to flowers that were not present prior to stress; Fig. 3). Faba bean is known to be capable of within‐plant resource redistribution, to maintain yield following changes in fertilization (Suso et al. 1996). Whole plant yield was reduced and yield allocation shifted at intermediate temperatures, rather than causing direct damage, intermediate temperature treatments may have accelerated plant development and limited time for grain development (González et al. 2011). Yield distributions of plants exposed to the two hottest temperature treatments (30 °C and 34 °C) showed a response more symptomatic of heat stress, with large yield reductions at lower nodal positions where flowers were present prior to stress. Reductions in yield at these particular nodal positions are of course an artefact of the timing of stress; reductions in yield may be expected to occur wherever flowers are present at a susceptible stage of development. Furthermore, plants exposed to stress after some pods have set on low nodal positions may perhaps show lower overall yield reductions. While peak yield allocation on the primary stem showed high plasticity, full yield compensation was not achieved. Higher flowers on which yield compensation occurred may have sustained damage, although to a lesser extent than lower flowers, and pods developing at higher nodal positions face increased within‐plant competition for resources (Patrick and Stoddard 2010). The capacity of faba bean for within‐plant yield compensation by self‐fertilization may be further facilitated by beneficial interactions with additional cross‐pollination by insects (*e.g*. Free 1993) or improved resource uptake via rhizobial symbionts (*e.g*. Denton et al. 2013). These findings suggest studies that measure damage to subsections of a plant may overestimate the effects of heat stress, that classical threshold models for crop stress responses (*e.g*. Craufurd et al. 2013) may be of limited applicability to indeterminate crops and that breeding faba beans for determinate growth habit will reduce resilience to stress.

In summary, faba bean yield was reduced by heat stress within the temperature range known to elicit yield reductions in other crop species, and as in other species, it is likely that reductions were due to gametophyte damage and consequent failure of fertilization. Due to their nature, it is difficult to predict how the frequency and magnitude of high temperature anomalies will change and consequently impact on food security, and this is complicated by projected changes in mean temperature, water availability and atmospheric CO_2_ levels (Porter et al. 2014). It is clear, however, that high temperature anomalies have already increased in both frequency and magnitude with climate change (Hansen et al. 2012), and they are likely to continue to do so (Kirtman et al. 2013). Faba bean is already notorious for low yield stability. The results of this study suggest that faba bean yield will become increasingly unstable with projected climate change and highlight the need to improve heat stress resilience of faba bean floral development and anthesis to promote future yield stability.

## Supporting information


**Table S1**. Summary of floral development scores measured on the primary stems of all experimental plants prior to temperature treatments.
**Table S2**. Summary of pollen germination data from a subset of plants sampled in year one.

## References

[jac12172-bib-0001] Ahmed, F. E. , A. E. Hall , and D. A. DeMason , 1992: Heat Injury during floral development in Cowpea (*Vigna unguiculata*,* Fabaceae*). Am. J. Bot. 79, 784–791.

[jac12172-bib-0002] Barber, H. M. , J. Carney , F. Alghabari , and M. J. Gooding , 2015: Decimal growth stages for precision wheat production in changing environments? Annal. Appl. Biol. 166, 355–371.

[jac12172-bib-0003] Bennell, M. R. , H. A. Cleugh , J. F. Leys , and D. Hein , 2007: The effect of hot dry wind on the pod set of faba bean (*Vicia faba*) cv. Fiord: a preliminary wind tunnel study. Aust. J. Exp. Agric. 47, 1468–1475.

[jac12172-bib-0004] Craufurd, P. Q. , V. Vadez , S. V. K. Jagadish , P. V. V. Prasad , and M. Zaman‐Allah , 2013: Crop science experiments designed to inform crop modeling. Agric. For. Meteorol. 170, 8–18.

[jac12172-bib-0005] Crawley, M. J. , 2013: The R Book. 2nd edn John Wiley & Sons Ltd, Chichester, UK.

[jac12172-bib-0006] Crépon, K. , P. Marget , C. Peyronnet , B. Carrouée , P. Arese , and G. Duc , 2010: Nutritional value of faba bean (*Vicia faba* L.) seeds for feed and food. Field. Crop. Res. 115, 329–339.

[jac12172-bib-0007] Dafni A., Kevan P. G., and Husband B. C., eds. 2005: Practical Pollination Biology. 1st edn Enviroquest Ltd, Cambridge, Ontario, Canada.

[jac12172-bib-0008] Denton, M. D. , D. J. Pearce , and M. B. Peoples , 2013: Nitrogen contributions from faba bean (*Vicia faba* L.) reliant on soil rhizobia or inoculation. Plant Soil, 365, 363–374.

[jac12172-bib-0009] Deryng, D. , D. Conway , N. Ramankutty , J. Price , and R. Warren , 2014: Global crop yield response to extreme heat stress under multiple climate change futures. Environ. Res. Lett. 9, 034011.

[jac12172-bib-0010] Flores, F. , S. Nadal , I. Solis , J. Winkler , O. Sass , F. L. Stoddard , W. Link , et al., 2012: Faba bean adaptation to autumn sowing under European climates. Agron. Sustain. Dev. 32, 727–734.

[jac12172-bib-0011] Free, J. B. , 1993: Insect Pollination of Crops. 2nd edn Academic Press Limited, London, UK.

[jac12172-bib-0012] González, F. G. , D. J. Miralles , and G. A. Slafer , 2011: Wheat floret survival as related to pre‐anthesis spike growth. J. Exp. Bot. 62, 4889–4901. 21705386 10.1093/jxb/err182

[jac12172-bib-0013] Gross, Y. , and J. Kigel , 1994: Differential sensitivity to high temperature of stages in the reproductive development of common bean (*Phaseolus vulgaris* L.). Field. Crop. Res. 36, 201–212.

[jac12172-bib-0014] Hamada, A. M. , 2001: Alteration in growth and some relevant metabolic processes of broad bean plants during extreme temperatures exposure. Acta Physiol. Plant 23, 193–200.

[jac12172-bib-0015] Hansen, J. , M. Sato , and R. Ruedy , 2012: Perception of climate change. Proc. Natl Acad. Sci. 109, E2415–E2423. 22869707 10.1073/pnas.1205276109 PMC3443154

[jac12172-bib-0016] Hedhly, A. , 2011: Sensitivity of flowering plant gametophytes to temperature fluctuations. Environ. Exp. Bot. 74, 9–16.

[jac12172-bib-0017] Kambal, A. E. , 1969: Flower drop and fruit set in field beans, *Vicia faba* L. J. Agric. Sci. 72, 131–138.

[jac12172-bib-0018] Khan, H. R. , J. G. Paull , K. H. M. Siddique , and F. L. Stoddard , 2010: Faba bean breeding for drought‐affected environments: A physiological and agronomic perspective. Field. Crop. Res. 115, 279–286.

[jac12172-bib-0019] Kirtman, B. , S. B. Power , A. J. Adedoyin , G. J. Boer , R. Bojariu , I. Camilloni , and F. Doblas‐Reyes , et al. 2013 Near‐term Climate Change: Projections and Predictability In Stocker T. F., Qin D., Plattner G.‐K., Tignor M., Allen S. K., Boschung J., and Nauels A., et al., eds. Climate Change 2013: The Physical Science Basis. Contribution of Working Group I to the Fifth Assessment Report of the Intergovernmental Panel on Climate Change, pp. 953–1028. Cambridge University Press, Cambridge, United Kingdom and New York, NY, USA.

[jac12172-bib-0020] Köpke, U. , and T. Nemecek , 2010: Ecological services of faba bean. Field. Crop. Res. 115, 217–233.

[jac12172-bib-0021] Lancashire, P. D. , H. Bleiholder , T. Boom , P. Langelüddeke , R. Stauss , E. Weber , and A. Witzenberger , 1991: A uniform decimal code for growth stages of crops and weeds. Annal. Appl. Biol. 119, 561–601.

[jac12172-bib-0022] Luo, Q. , 2011: Temperature thresholds and crop production: a review. Clim. Change. 109, 583–598.

[jac12172-bib-0023] McDonald, G. K. , and G. M. Paulsen , 1997: High temperature effects on photosynthesis and water relations of grain legumes. Plant Soil 196, 47–58.

[jac12172-bib-0024] Monterroso, V. A. , and H. C. Wien , 1990: Flower and Pod Abscission Due to Heat Stress in Beans. J. Am. Soc. Hortic. Sci. 115, 631–634.

[jac12172-bib-0025] Oney, S. , and S. Tabur , 2013: Cytogenetical and Molecular Responses of Exogenous Potassium Sulphate for Tolerance to Extreme Temperatures in *Vicia faba* L. J. Pure Appl. Microbiol. 7, 663–670.

[jac12172-bib-0026] Osborne, J. L. , C. S. Awmack , S. J. Clark , I. H. Williams , and V. C. Mills , 1997: Nectar and flower production in *Vicia faba* L. (field bean) at ambient and elevated carbon dioxide. Apidologie 28, 43–55.

[jac12172-bib-0027] Patrick, J. W. , and F. L. Stoddard , 2010: Physiology of flowering and grain filling in faba bean. Field. Crop. Res. 115, 234–242.

[jac12172-bib-0028] Porch, T. , and M. Jahn , 2001: Effects of high‐temperature stress on microsporogenesis in heat‐sensitive and heat‐tolerant genotypes of *Phaseolus vulgaris* . Plant, Cell Environ. 24, 723–731.

[jac12172-bib-0029] Porter, J. R. , L. Xie , A. J. Challinor , K. Cochrane , S. M. Howden , M. M. Iqbal , and D. B. Lobell , et al. 2014 Food security and food production systems In Field C. B., Barros V. R., Dokken D. J., Mach K. J., Mastrandrea M. D., Bilir T. E., and Chatterjee M., et al., eds. Climate Change 2014: Impacts, Adaptation, and Vulnerability. Part A: Global and Sectoral Aspects. Contribution of Working Group II to the Fifth Assessment Report of the Intergovernmental Panel on Climate Change, pp. 485–533. Cambridge University Press, Cambridge, United Kingdom and New York, NY, USA.

[jac12172-bib-0030] Prasad, P. V. V. , K. J. Boote , L. H. Allen , and J. M. G. Thomas , 2003: Super‐optimal temperatures are detrimental to peanut (*Arachis hypogaea* L.) reproductive processes and yield at both ambient and elevated carbon dioxide. Glob. Change Biol. 9, 1775–1787.

[jac12172-bib-0031] Pretty, J. , and Z. P. Bharucha , 2014: Sustainable intensification in agricultural systems. Ann. Bot. 114, 1571–1596. 25351192 10.1093/aob/mcu205 PMC4649696

[jac12172-bib-0032] R Core Team , 2014: R: A Language and Environment for Statistical Computing. R Foundation for Statistical Computing, Vienna, Austria URL [http://www.R-project.org/].

[jac12172-bib-0033] Siebert, S. , and F. Ewert , 2014: Future crop production threatened by extreme heat. Environ. Res. Lett. 9, 041001.

[jac12172-bib-0034] Stelling, D. , W. Link , and E. Ebmeyer , 1994: Factors determining the performance of synthetics in *Vicia faba* L. Euphytica 75, 85–93.

[jac12172-bib-0035] Suso, M. J. , M. T. Moreno , F. Mondragao‐Rodrigues , and J. I. Cubero , 1996: Reproductive biology of *Vicia faba*: role of pollination conditions. Field. Crop. Res. 46, 81–91.

[jac12172-bib-0036] Suzuki, K. , T. Tsukaguchi , H. Takeda , and Y. Egawa , 2001: Decrease of pollen stainability of green bean at high temperatures and relationship to heat tolerance. J. Am. Soc. Hortic. Sci. 126, 571–574.

[jac12172-bib-0037] Young, L. W. , R. W. Wilen , and P. C. Bonham‐Smith , 2004: High temperature stress of Brassica napus during flowering reduces micro‐ and megagametophyte fertility, induces fruit abortion, and disrupts seed production. J. Exp. Bot. 55, 485–495. 14739270 10.1093/jxb/erh038

[jac12172-bib-0038] Zinn, K. E. , M. Tunc‐Ozdemir , and J. F. Harper , 2010: Temperature stress and plant sexual reproduction: uncovering the weakest links. J. Exp. Bot. 61, 1959–1968. 20351019 10.1093/jxb/erq053 PMC2917059

