# Supplementary material for: Susceptibility of Faba Bean (Vicia faba L.) to Heat Stress During Floral Development and Anthesis
Source: J Agron Crop Sci. 2016 Mar 21;202(6):508–17. doi: 10.1111/jac.12172 (PMC5763371; doi:10.1111/jac.12172)
Supplement: Supplementary file 1 — Table S1. Summary of floral development scores measured on the primary stems of all experimental plants prior to temperature treatments. Table S2. Summary of pollen germination data from a subset of plants sampled in year one. [file JAC-202-508-s001.pdf]

Supplementary Information

Table S1: Summary of floral development scores measured on the primary stems of all experimental plants prior to temperature treatments.

**Heat**	**No. floral nodes on primary stem with flowers present (stage ≥ 1); mean ± SD**				**No. floral nodes on primary stem with flowers open (stage ≥ 7); mean ± SD**			
Rep.	1	2	3	4	1	2	3	4
**18**	4.5 ± 2.0	5.5 ± 1.2	5.7 ± 2.1	3.6 ± 2.2	1.4 ± 1.1	0.8 ± 1.5	2.0 ± 1.2	0.4 ± 0.7
**22**	5.6 ± 1.5	6.6 ± 1.3	6.3 ± 1.6	3.7 ± 2.5	1.6 ± 1.2	2.2 ± 1.9	2.9 ± 0.8	0.4 ± 0.9
**26**	6.1 ± 1.3	6.2 ± 1.6	5.6 ± 1.5	4.1 ± 2.2	2.3 ± 1.1	1.7 ± 1.6	2.3 ± 1.2	0.4 ± 0.7
**30**	6.2 ± 1.2	5.7 ± 1.2	6.4 ± 1.2	3.8 ± 1.9	2.2 ± 1.1	1.0 ± 1.1	2.4 ± 0.8	0.5 ± 0.8
**34**	5.1 ± 1.0	6.5 ± 1.0	6.1 ± 1.9	3.6 ± 1.9	1.4 ± 1.1	1.9 ± 1.4	2.4 ± 0.8	0.2 ± 0.4

Table S2: Summary of pollen germination data from a subset of plants sampled in year one.

**Heat**	**Proportion of germination per flower per plant**			**Flowers sampled**			**No. germinated pollen grains**			**No. total pollen grains**		
Rep.	1	2	3	1	2	3	1	2	3	1	2	3
**18**	0.771	0.605	0.765	23	24	21	1109	651	661	1355	1075	863
**22**	0.777	0.625	0.835	32	26	14	1233	694	577	1801	1355	699
**26**	0.680	0.541	0.887	25	25	19	1272	469	651	1984	1098	743
**30**	0.472	0.526	0.604	15	19	22	321	408	422	646	1152	649
**34**	0.079	0.071	0.089	26	7	3	138	25	5	1894	464	99
